# Supplementary material for: Ethylene-mediated nitric oxide depletion pre-adapts plants to hypoxia stress
Source: Nat Commun. 2019 Sep 5;10:4020. doi: 10.1038/s41467-019-12045-4 (PMC6728379; doi:10.1038/s41467-019-12045-4)
Supplement: Supplementary file 1 — Supplementary Information [file 41467_2019_12045_MOESM1_ESM.pdf]

Supplementary Information

Ethylene-mediated nitric oxide depletion pre-adapts plants to hypoxia stress

Sjon Hartman[1], Zeguang Liu[1], Hans van Veen[1], Jorge Vicente[2], Emilie Reinen[1], Shanice Martopawiro[1], Hongtao Zhang[3], Nienke van Dongen[1], Femke Bosman[1], George W. Bassel[4], Eric J.W. Visser[5], Julia Bailey-Serres[1,6], Frederica L. Theodoulou[3], Kim H. Hebelstrup[7], Daniel J. Gibbs[4], Michael J. Holdsworth[2]*, Rashmi Sasidharan[1]* and Laurentius A.C.J. Voesenek[1]*

[1.] Plant Ecophysiology, Institute of Environmental Biology, Utrecht University, Padualaan 8, 3584 CH, Utrecht, The Netherlands
[2.] School of Biosciences, University of Nottingham, Loughborough, LE12 5RD, United Kingdom
[3.] Plant Sciences Department, Rothamsted Research, Harpenden, AL5 2JQ, United Kingdom
[4.] School of Biosciences, University of Birmingham, Edgbaston B15 2TT, United Kingdom
[5.] Department of Experimental Plant Ecology, Institute for Water and Wetland Research, Radboud University Nijmegen, 6525 AJ, Nijmegen, the Netherlands
[6.] Botany and Plant Sciences Department and Center for Plant Cell Biology, University of California, Riverside, California 92521, United States
[7.] Department of Molecular Biology and Genetics, Aarhus University, Forsøgsvej 1, DK-4200 Slagelse, Denmark

This Supplementary Information file contains:

Supplementary Figures 1-10
Supplementary Table 1 (Genotyping primers)
Supplementary Table 2 (RT-qPCR primers)

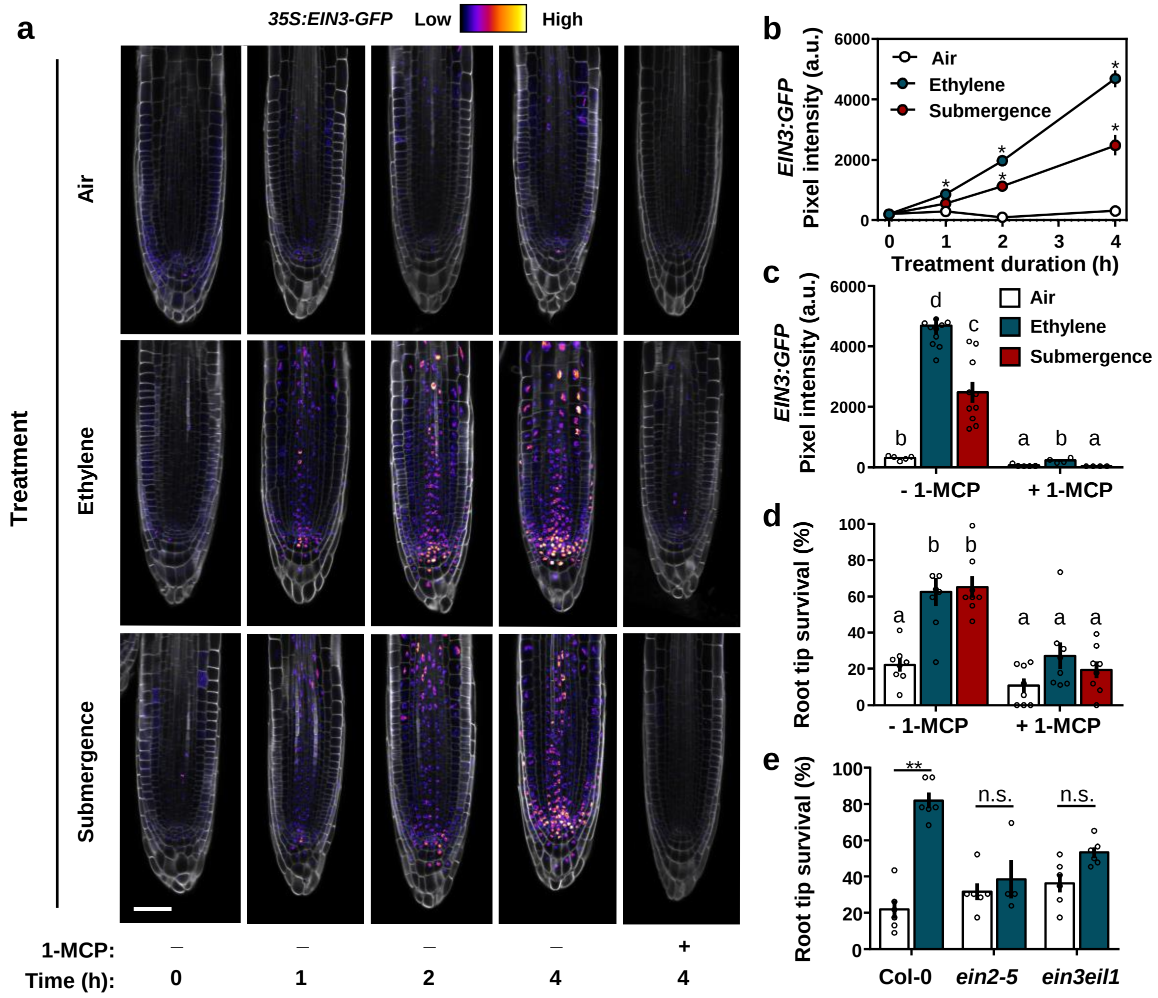

Supplementary Figure 1. Ethylene signalling mediates early submergence responses towards hypoxia acclimation

(a) Representative confocal images and quantification (**b, c**) of protein stability and localization of the ethylene master regulator EIN3, using the *35S:EIN3-GFP* (*ein3eil1* double mutant background) signal in Arabidopsis root tips. Seedling were treated for up to 4 hours with air (white), ~5μll⁻¹ ethylene (blue) or submergence (red), either in combination with or without a pre-treatment of ethylene action inhibitor 1-MCP at the 4 hour time-point. Cell walls were visualized using Calcofluor White stain (scale bar= 50μm). Mean GFP pixel intensity inside the root tips was quantified using ICY imaging software. (b) Asterisks indicate a significant difference from the air mean per time-point ($p<0.05$, ANOVA with planned comparisons, Tukey's HSD correction for multiple comparisons, n=5-12 roots). (c) Samples without 1-MCP are the same as in (b) at t=4h. Statistically similar groups are indicated using the same letter ($p<0.05$, 2-way ANOVA, Tukey's HSD, n=5-11 roots). **(d, e)** Seedling root tip survival of Col-0 after 4 hours of pre-treatment with air (white), ~5μll⁻¹ ethylene (blue) or submergence (red), either in combination with or without a pre-treatment of ethylene action inhibitor 1-MCP (d), or Col-0 and two ethylene signalling pathway loss-of-function mutants (e) followed by 4 hours of hypoxia and 3 days of recovery. Values are relative to control (normoxia) plants. Statistically similar groups are indicated using the same letter in (d). Asterisks indicate significant differences between air and ethylene in (e) ($p<0.05$, Generalized linear model with negative binomial error structure, (d) n=8 rows of ~23 seedlings; (e) n=4-6 rows of ~46 seedlings). Error bars are SEM, experiments were replicated at least 2 times.

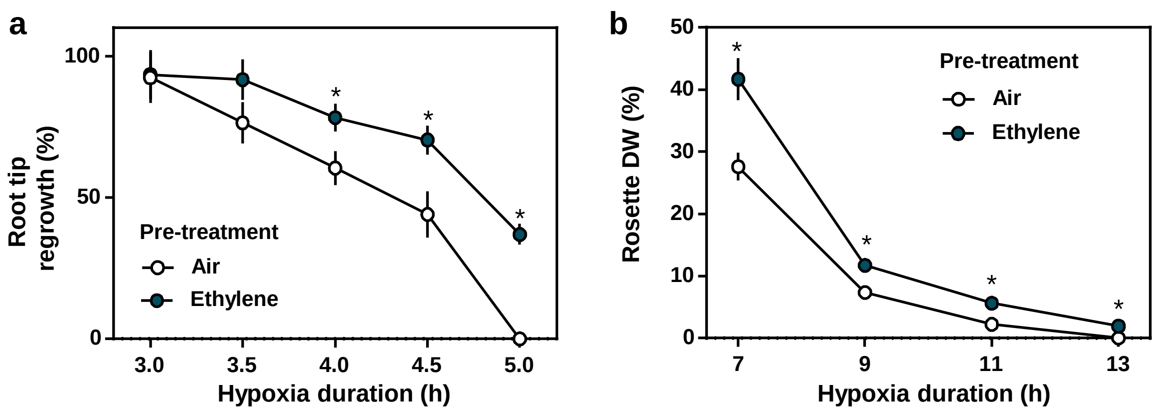

Supplementary Figure 2. Ethylene pre-treatment enhances plant performance of recovering tissues after subsequent hypoxia

(a) Seedling root tip regrowth capacity of surviving roots after 4 hours of pre-treatment with air (white) or ~5µll⁻¹ ethylene (blue) followed by hypoxia and 3 days of recovery. Values are relative to control (normoxia) plants. Asterisks indicate significant differences between air and ethylene at given time point (*p<0.05, Student's *t* test, n=4-8 rows of 23 seedlings for survival, n= 5-35 surviving roots for regrowth). **(b)** Rosette dry weight (DW) of adult Col-0 plants after 4 hours of pre-treatment with air (white) or ~5µll⁻¹ ethylene (blue) followed by hypoxia and 7 days of recovery. DW was measured only from surviving plants. Values are relative to control (normoxia) plants. Asterisks indicate significant differences between air and ethylene at given time point (*p<0.05, Student's *t* test, n=30 plants). Error bars are SEM, experiments were replicated at least 3 times.

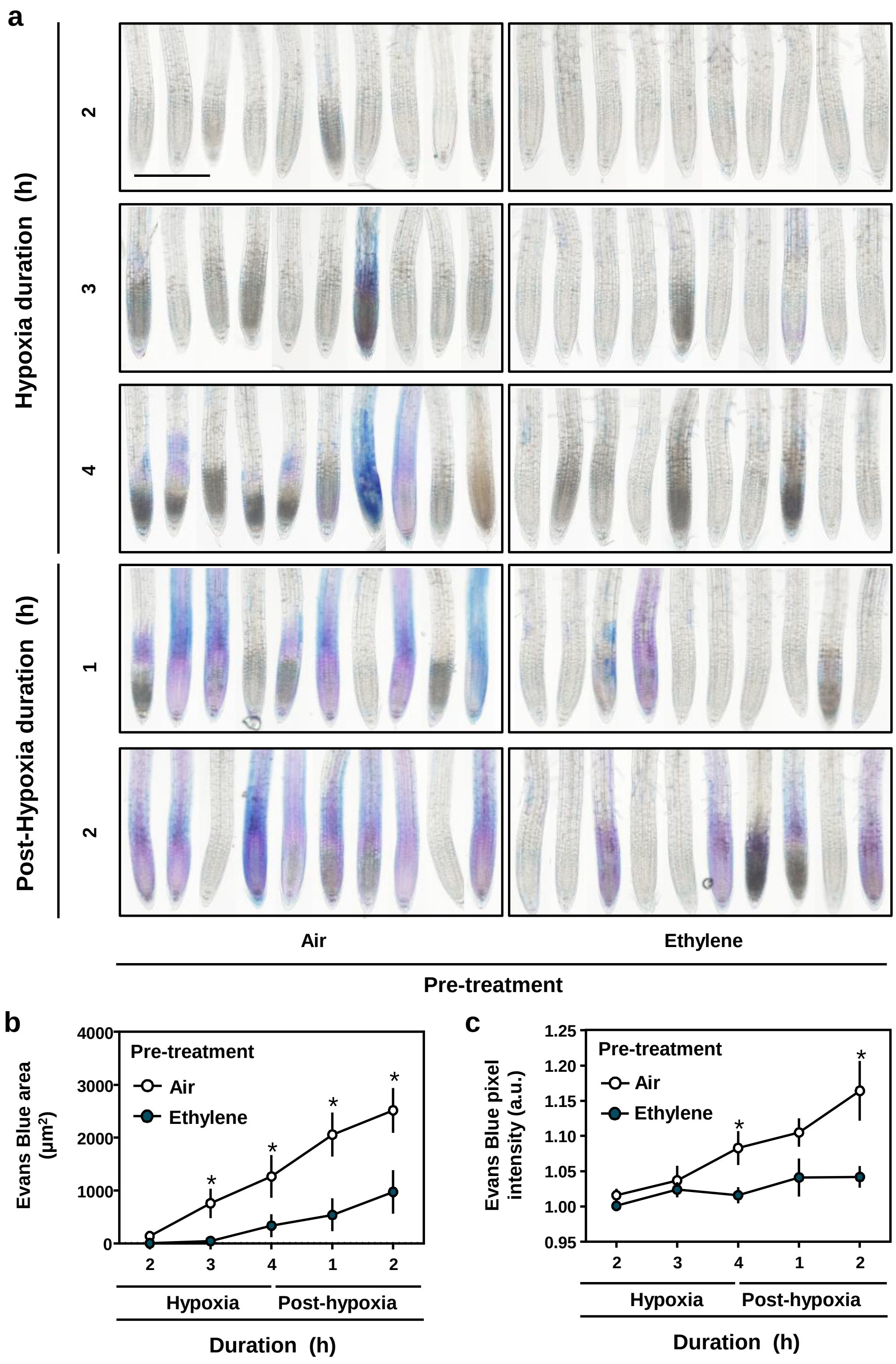

Supplementary Figure 3. Ethylene pre-treated seedlings show reduced cell damage in root tips during subsequent hypoxia and recovery treatments

(a) Representative light microscopy images of Evans blue staining for impaired cell membrane integrity in seedling root tips after 4 hours of pre-treatment with air or ~5μll^{-1} ethylene followed by 2-4h hypoxia and 1-2h of recovery (scale bar = 2mm). **(b, c)** Quantification of the area (b) and pixel intensity (c) of Evans blue staining in seedling root tips after 4 hours of pre-treatment with air (white) or ~5μll^{-1} ethylene (blue) followed by 2-4h hypoxia and 1-2h of recovery. Asterisks indicate significant differences between air and ethylene at given time point (Error bars are SEM, *p<0.05, Student's *t* test, n=10 root tips). Experiments were replicated at least 3 times.

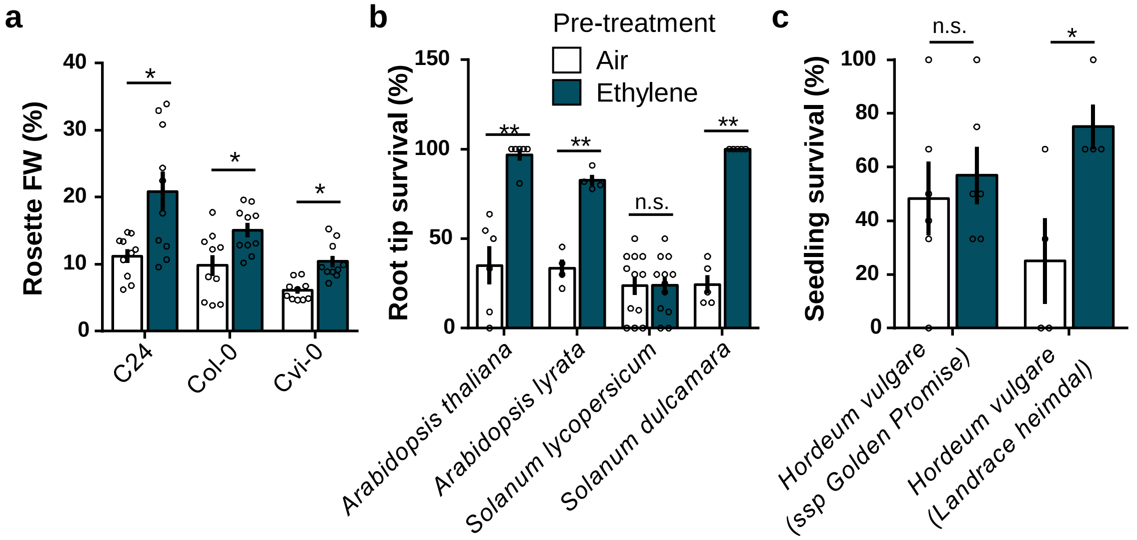

Supplementary Figure 4. Ethylene-induced hypoxia tolerance is conserved within Arabidopsis thaliana accessions and shows variation between other plant species

(a) Relative rosette fresh weight (FW) of adult Arabidopsis accessions C24, Col-0 and Cvi-0 plants after 4 hours of pre-treatment with air (white) or ~5μll⁻¹ ethylene (blue) followed by 9 hours of hypoxia and 7 days of recovery. FW was measured only from survived plants (Error bars are SEM, *p<0.05, Student's *t* test, n=10 plants). **(b)** Root tip survival of 4 different plants species after 4 hours of pre-treatment with air (white) or ~5μll⁻¹ ethylene (blue) followed by 4 hours of hypoxia and 3 days of recovery Asterisks indicate significant differences between air and ethylene (Error bars are SEM, **p<0.01, Generalized linear model with negative binomial error structure, n=4-12 rows of 10-46 seedlings depending on species). **(c)** Plant survival of 2 different varieties of Barley (*Hordeum vulgare*) seedlings after 4 hours of pre-treatment with air (white) or ~5μll⁻¹ ethylene (blue) followed by 20 hours of hypoxia and 3 days of recovery. Asterisks indicate significant differences between air and ethylene (Error bars are SEM, *p<0.05, Generalized linear model with negative binomial error structure, n=4-6 replicates containing 3 seedlings). Experiments were replicated at least 2 times, except for a, which was only performed once.

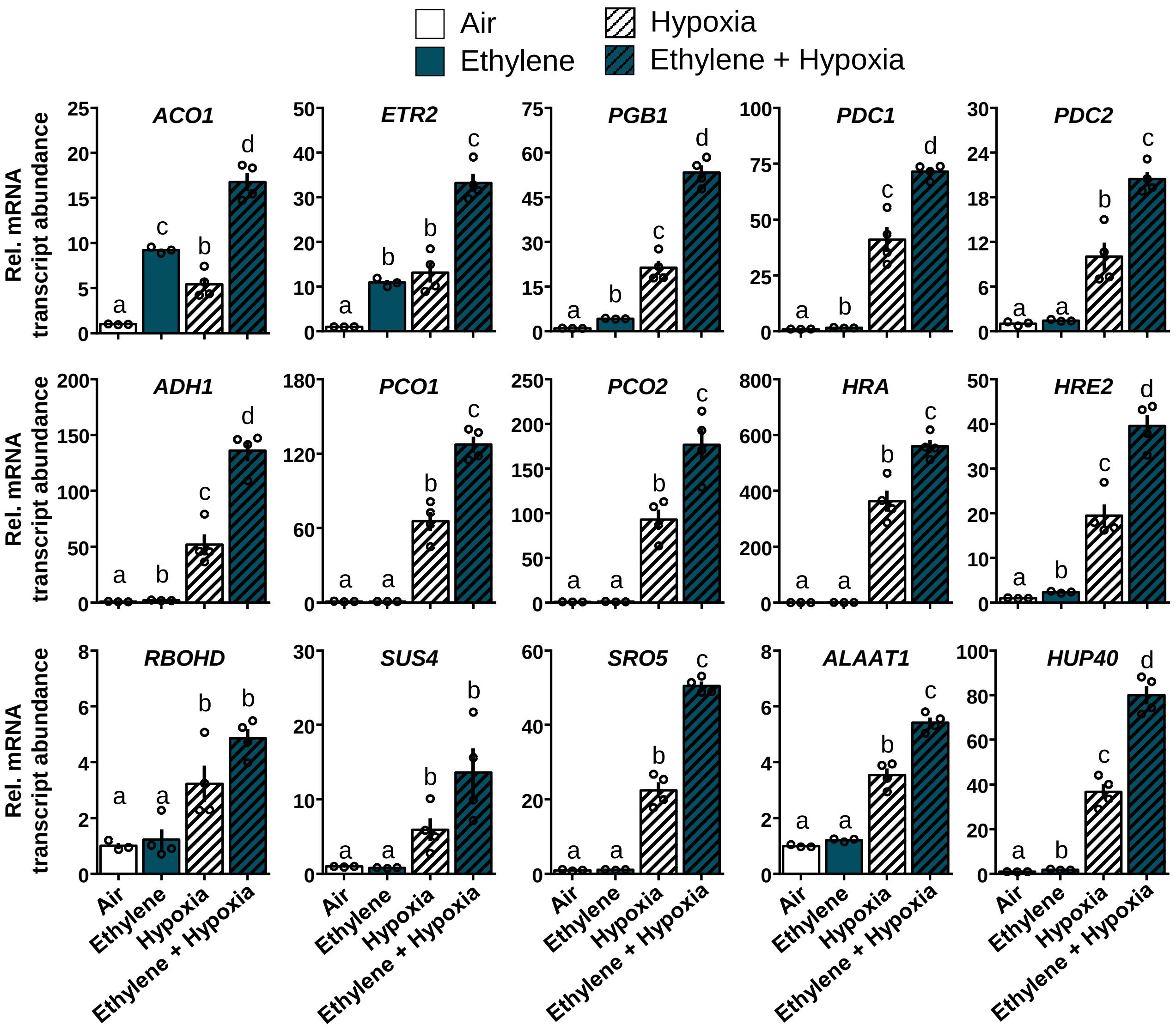

Supplementary Figure 5. Ethylene pre-treatment augments hypoxia adaptive gene transcripts upon hypoxia

Relative mRNA transcript abundance of 15 hypoxia adaptive genes in seedling root tips of Col-0 after 4 hours of pre-treatment with air (white) or ~5μll^{-1} ethylene (blue), followed by (4h) hypoxia (dashed stripes). Values are relative to Col-0 air treated samples. Different letters indicate significant differences (Error bars are SEM, $p<0.05$, 1-way ANOVA, Tukey's HSD, n=3-4 replicates of ~400 root tips). Experiments were replicated at least 2 times.

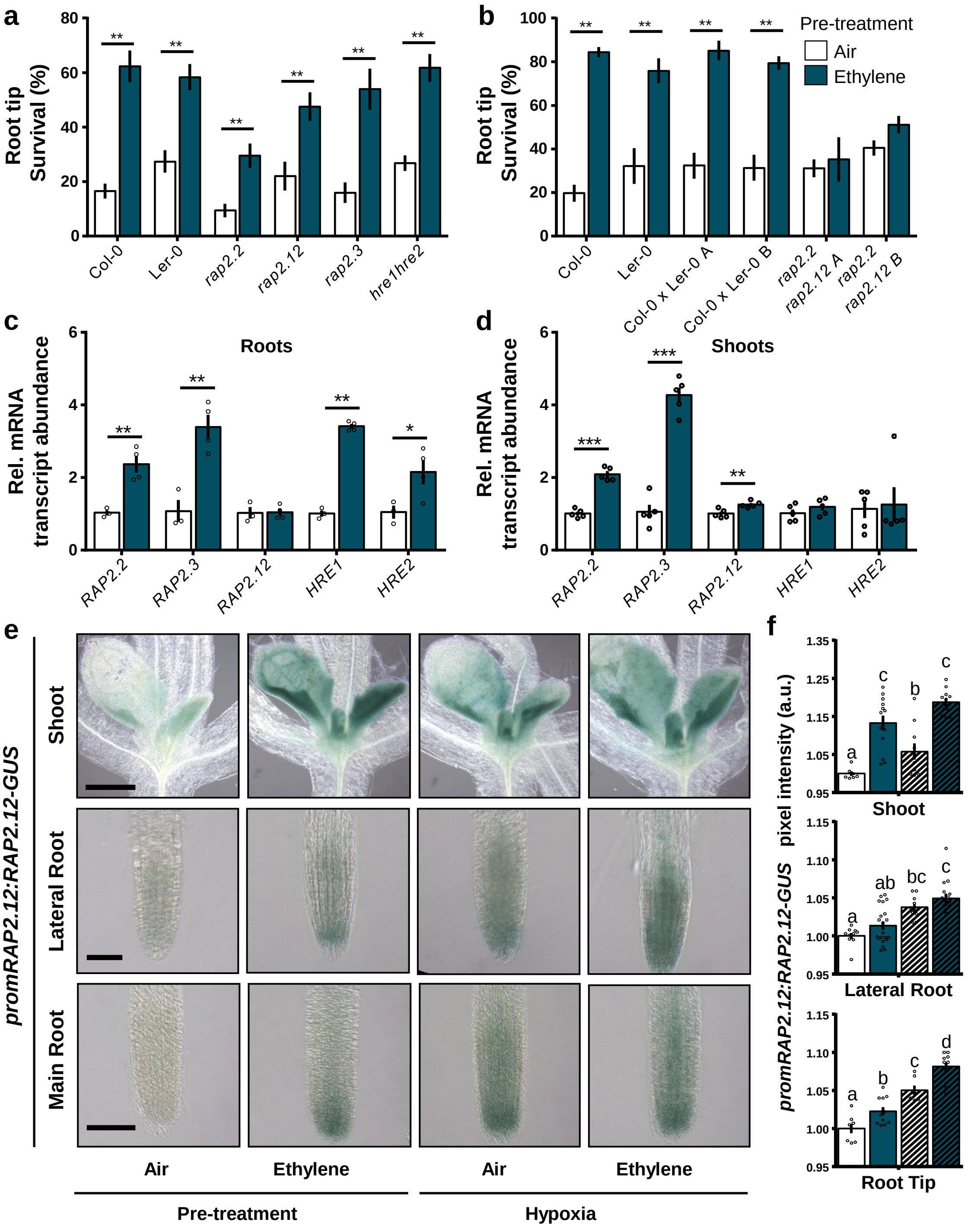

Supplementary Figure 6. Involvement and regulation of ERFVIIs during ethylene-induced hypoxia tolerance
(a, b) Seedling root tip survival of Col-0, Ler-0, ERFVII mutants *rap2.2* (Ler-0 background), *rap2.12*, *rap2.3* and *hre1hre2* (Col-0 background) in a, and Col-0, Ler-0, 2 Col-0 x Ler-0 WT crosses and ERFVII double mutants *rap2.2rap2.12* (2 independent lines in Col-0 x Ler-0 background) in b, after 4 hours of pre-treatment with air (white) or ~5μll⁻¹ ethylene (blue) followed by 4 hours of hypoxia and 3 days of recovery. Values are relative to control (normoxia) plants. Asterisks indicate significant differences between air and ethylene (Error bars are SEM, **p<0,01, Generalized linear model with negative binomial error structure, n=4-21 rows consisting of ~23 seedlings for a, n=8 rows consisting of ~23 seedlings for b). **(c, d)** Relative mRNA transcript abundance of all 5 ERFVIIs in root tips of Col-0 seedlings (c) and adult rosettes (d) after air (white) or ~5μll⁻¹ ethylene (blue) treatments (4h). Asterisks indicate significant differences between air and ethylene (Error bars are SEM,*p<0.05, **p<0.01, ***p<0.001, Generalized linear model with negative binomial error structure, n=3-4 replicates containing ~400 root tips for c, n=5 replicates of 2 rosettes for d). **(e, f)** Representative DIC microscopy images (e) and quantification (f) of *promRAP2.12::RAP2.12-GUS* in seedling shoots, lateral roots and main root tips after treatments with air (white) or ~5μll⁻¹ ethylene (blue) or subsequent (4h) hypoxia (dashed stripes). Scale bars; shoot = 200μm, lateral root = 60μm, main root = 100μm. Values are relative to air treated samples. Statistically similar groups are indicated using the same letter per tissue (Error bars are SEM, p<0.05, 1-way ANOVA, Tukey's HSD, n=5-20 replicates). Experiments were replicated at least 2 times.

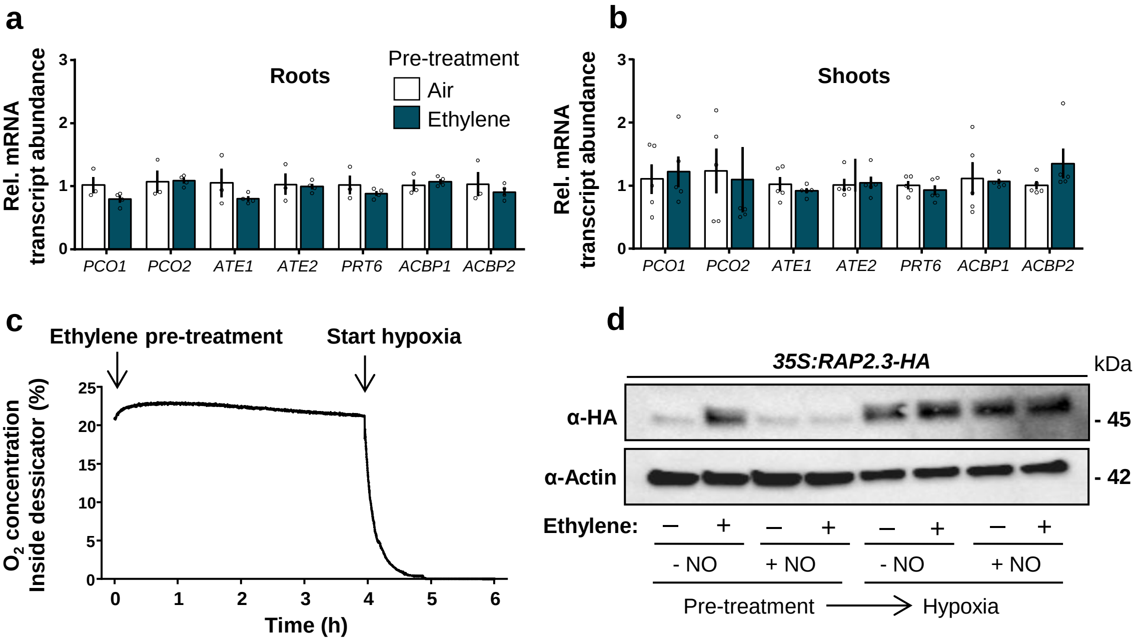

Supplementary Figure 7. The effects of ethylene on processes known to mediate ERFVII stability
(a, b) Relative mRNA transcript abundance of genes coding for enzymes involved in the PRT6 N-degron pathway or RAP2.12-sequestering Acyl-CoA binding proteins *ACBP1* and *ACBP2* in root tips of Col-0 seedlings (a) and adult rosettes (b) after 4 hours of treatment with air (white) or ~5μll^{-1} ethylene (blue). Values are relative to Col-0 air treated samples. No significant differences were found between air and ethylene (Error bars are SEM, Student's t test, n=3-4 replicates containing ~400 root tips for a, n=5 replicates of 2 rosettes for b). **(c)** Levels of molecular oxygen measured over time at the outflow of the desiccators during the ethylene pre-treatment and subsequent hypoxia treatments in this study. Oxygen levels generally reached <0.00% between 40 and 50 minutes of flushing the desiccators with humidified 99.996% N_2 at a rate of 2l min^{-1}. **(d)** RAP2.3 protein levels in *35S::MC-RAP2.3-HA* seedlings (Col-0 background) after air and ethylene pre-treatments (4h), combined with or without an additional NO pulse and subsequent hypoxia (4h). Experiments were replicated at least 2 times, except for d, in which the hypoxia treatment after NO manipulation was only performed once.

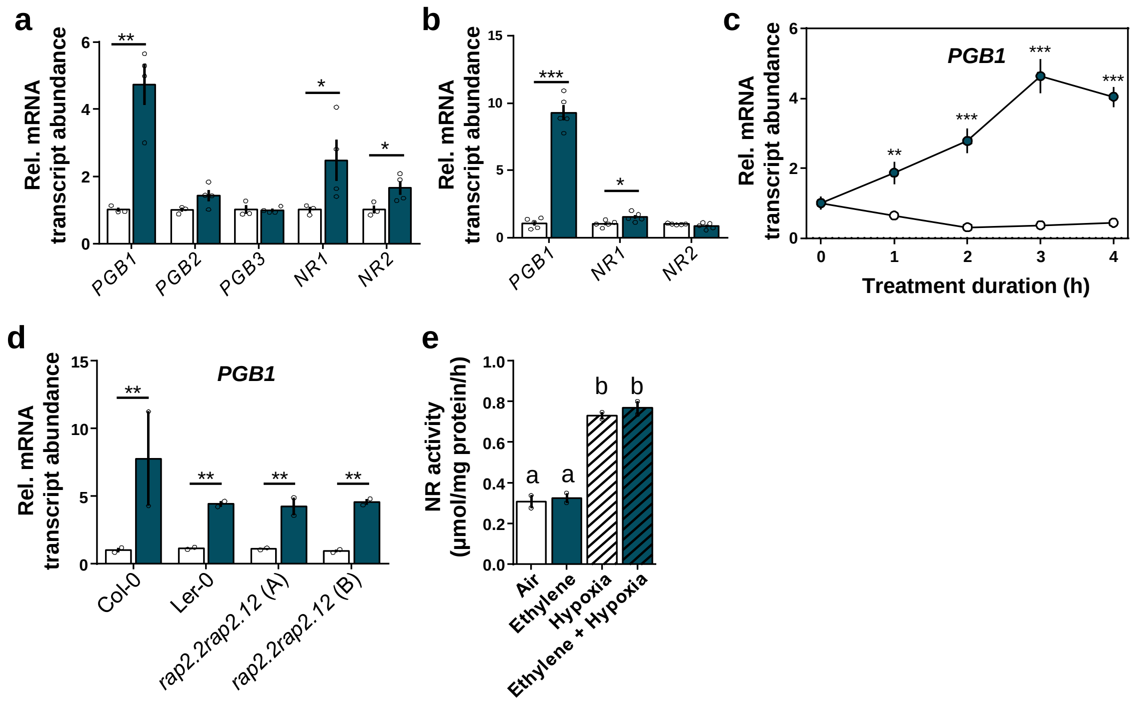

Supplementary Figure 8. The effects of ethylene on *PHYTOGLOBIN* transcript abundance and *NITRATE REDUCTASE* transcript abundance and activity

(a, b) Relative mRNA abundance of genes coding for enzymes involved in NO metabolism in seedling root tips (a) and adult rosettes (b) of Col-0 plants after 4 hours of treatment with air (white) or ~5µll⁻¹ ethylene (blue). Values are relative to Col-0 air treated samples. Asterisks indicate significant differences between air and ethylene (***p<0.001, **p<0.01, *p<0.05 Student's *t* test, n=3-4 replicates of ~400 root tips in a, n=5 replicates of 2 rosettes in b). **(c)** Relative *PGB1* mRNA transcript abundance in rosettes of Col-0 plants during 4 hours of treatment with air (white) or ~5µll⁻¹ ethylene (blue). Asterisks indicate significant differences between air and ethylene (***p<0.001, ANOVA with planned comparisons, Tukey's HSD correction for multiple comparisons, n=5 replicates of 2 rosettes). **(d)** Relative *PGB1* mRNA transcript abundance in seedlings of Arabidopsis Col-0 and Ler-0 WT, and 2 double *rap2.2rap2.12* mutants (Col-0 x Ler-0 background) after 4 hours of treatment with air (white) or ~5µll⁻¹ ethylene (blue). Values are relative to Col-0 air treated samples. Asterisks indicate significant differences between air and ethylene (**p<0.01, ANOVA with planned comparisons, Tukey's HSD correction for multiple comparisons, n=2 replicates of ~400 root tips). **(e)** Nitrate reductase activity in whole Col-0 WT seedlings after 4 hours of pre-treatment with air (white) or ~5µll⁻¹ ethylene (blue), followed by (4h) hypoxia (dashed stripes). Statistically similar groups are indicated using the same letter (p<0.05, 1-way ANOVA, Tukey's HSD, n=2 replicates of ~200 seedlings). All error bars are SEM, experiments were replicated at least 2 times, except for e, which was only performed once.

a

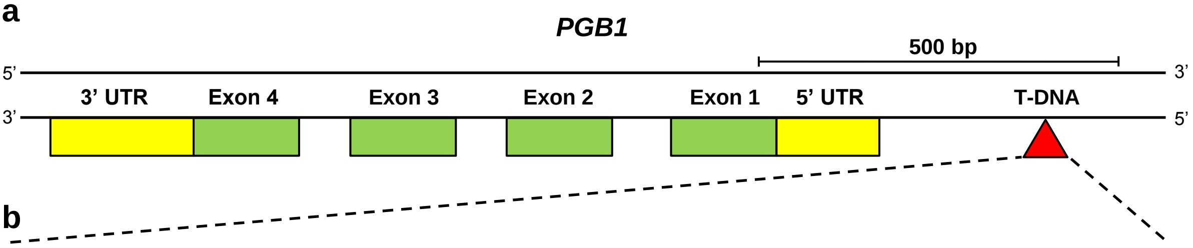

b

5’CATAATATTTCACAACCTCTAAATGATTTAAAGTATACAACTCTCTGTTTTGTAGATCTTACTCCAATTAT
GAAAAGTATCCTATATATAGGTTTTGATAAAAGACCCTTTTGTGTTACCGAAGAGACAGTTCTTGATAAGC
AAGTTTAAGATAAAGTTGCTTGGTTAAAAAGTGAAACCATGTGCTCTGTACTGGTAATGGAACATAGTTTG
TAATGGTAGGCTTGACCAATTATTAGTCAAAACACTAAAGAAAAGTCTTTTAGATCTCTTTATCTCATTAAT
TTACTTATTTATGTATTGCGGTGTAAACAAATTGACGCTTAGACAACTTAATAACACATTGCGGACGTTTTT
AATGTACTGGGGTGGTTTTTCTTTTCACCAGTGAGACGGGCAACAGCTGATTGCCCTTCACCGCCTGGC
CCTGAGAGAGTTGCAGCAAGCGGTCCACGCTGGTTTGCCCCAGCAGGCGAAGATCCTGTTTGATGGTG
GTTCCGAAATCGGCGAAATTAAAGAAAGGGCCGTTTTTTTTGTTTGTTTTTTCGAGTTCATAGGGATGAA3’

Supplementary Figure 9. Identification of *pgb1-1* mutant line *SK_058388*

(a) Schematic map of genomic *PGB1* gene region including the 4 *PGB1* exons (green) and the location of the t-DNA insertion (red triangle) of *pgb1-1* line *SK_058388.* **(b)** Partial DNA sequencing reaction of *pgb1-1* aligned with genomic *PGB*1 gene region. The aligned native *PGB1* sequence (black) ends and the T-DNA sequence (red) starts exactly 300bp upstream of the *PGB1* start codon.

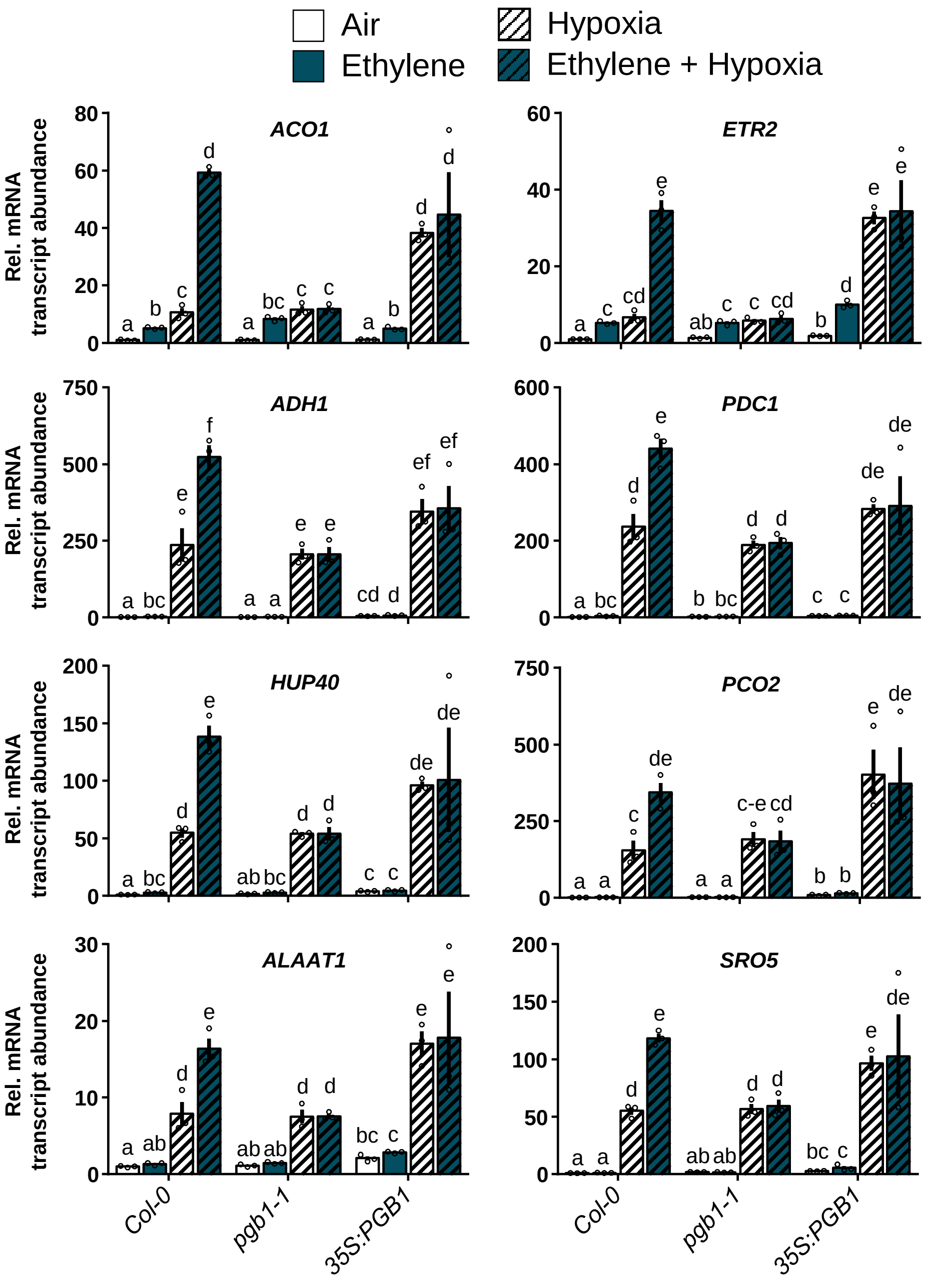

Supplementary Figure 10. Hypoxia adaptive gene expression in *PGB1* knock-down and over-expression lines

Relative mRNA transcript abundance of 8 hypoxia adaptive genes in seedling root tips of Col-0, *pgb1-1* and *35S:PGB1* after 4 hours of pre-treatment with air (white) or ~5μll^{-1} ethylene (blue), followed by (4h) hypoxia (dashed stripes). Values are relative to Col-0 air treated samples. Different letters indicate significant differences (Error bars are SEM, p<0.05, 2-way ANOVA, Tukey's HSD, n=3 replicates containing ~200 root tips).

Supplementary Table 1. List of genotyping primers used in this study

T-DNA lines	Primer info	Oligo sequence 5' → 3'	Additional info
rap2.2-5* *(AY201781)	WT FW	ccgcgtcactaacgagtttat	*Gasch et al., 2015* [20]
	WT REV	ctccactgggttttcctcttc	
	T-DNA REV	cgattaccgtatttatcccgt	
rap2.12-2* *(SAIL_1215_H10)	WT FW	tcttcgattttgacgctgagt	*Gasch et al., 2015* [20]
	WT REV	agggtttgcaccattgtcctgag	
	T-DNA REV	gaatttcataaccaatctcgatacac	
rap2.3-1* *(SAIL_1031_D10)	WT FW	atgtgtggcggtgctattatt	*Gibbs et al., 2014* [14]
	WT REV	ttactcatacgacgcaatgac	
	T-DNA REV	gaatttcataaccaatctcgatacac	
hre1* *(SALK_039484)	WT FW	ttacagacagtggcgaaatca	*Gibbs et al., 2014* [14]
	WT REV	tcaggaccatagacccatgt	
	T-DNA REV	attttgccgatttcggaac	
hre2* *(SALK_052858)	WT FW	tgcaaaaggttatagagcacac	*Gibbs et al., 2014* [14]
	WT REV	ggcaaccggaatctgataga	
	T-DNA REV	attttgccgatttcggaac	
prt6-1* *(SAIL_1278_H11)	WT FW	ggcagaaacatccctgaaag	*Gibbs et al., 2011* [12]
	WT REV	gcagcacaacactggagaag	
	T-DNA REV	gaatttcataaccaatctcgatacac	
pgb1-1* *(SALK_058388)	WT FW	aagtgttacgtgagactacgact	This paper
	WT REV	cttcgttgttggtgcaatctca	
	T-DNA REV	attttgccgatttcggaac	
eil1-1	WT FW	tttgatcgtaatggtccagc	*Alonso et al., 2003* [39]
	WT REV	attttgctgtgaggacactg	
	Transp.REV	gtcggtccccacacttctata	
**Transgenic lines**	**Primer info**	**Oligo sequence 5' → 3'**	**Additional info:**
35S:PGB1	35S:FW	ggaagttcatttcatttggagagg	Kanamycin Resistance
	PGB1 REV	tgacactccaagacttcactaca	*Hebelstrup et al., 2006* [40]
35S:RAP2.3-HA	35S:FW	ggaagttcatttcatttggagagg	Basta Resistance
	RAP2.3 REV	taatcggaaataatagcaccgcc	*Gibbs et al., 2014* [14]
35S:EIN3-GFP	35S:FW	ggaagttcatttcatttggagagg	Kanamycin resistance<br>In *ein3eil1-1* background
	EIN3 REV	atgcttgataaccgcagtca	*Xie et al., 2015* [7]
35S:RAP2.12-GFP	35S:FW	ggaagttcatttcatttggagagg	Kanamycin Resistance
	RAP2.12 REV	agggtttgcaccattgtcctgag	*Licausi et al., 2011* [13]
35S:δ13-RAP2.12-GFP	35S:FW	ggaagttcatttcatttggagagg	Kanamycin Resistance
	RAP2.12 REV	agggtttgcaccattgtcctgag	*Licausi et al., 2011* [13]
promRAP2.12:RAP2.12-GUS	RAP2.12 FW	actgaatgggacgcttcactgg	Hygromycin Resistance
	GUS REV	ccatcagcacgttatcgaat	This paper
**Other**	**Primer info**	**Oligo sequence 5' → 3'**	**Additional info:**
ein2-5	WT FW	cgctcattccagtggtcttt	7bp deletion
	WT REV	tggtatattccgtctgcacca	*Alonso et al., 1999* [38]
ein3	WT FW	aggaggatgtggagagacaa	G to A substitution at nt1598
	WT REV	atgcttgataaccgcagtca	*Alonso et al., 2003* [39]

Supplementary Table 2. List of RT-qPCR primers used in this study

Target gene	AT code	Primer name	Oligo sequence 5' → 3'
ACBP1	*AT5G53470*	ACBP1_FW	TGGAGATGCGTTATTGTGA
		ACBP1_R	GCGAGAAGGTAAGCGAAG
ACBP2	*AT4G27780*	ACBP2_FW	GTGAGGCGGATTCGCTTGT
		ACBP2_R	TGCGGCGGCGGTAGTC
ACO1	*AT2G19590*	ACO1_FW	CCTCAGATGCAGATTGGGAAAGC
		ACO1_R	TCATCCATCGTCTTGCTGAGTTCC
ADH1	*AT1G77120*	ADH1_FW	GGTCTTGGTGCTGTTGGTTT
		ADH1_R	CTCAGCGATCACCTGTTGAA
APT1	*AT1G27450*	APT1_FW	AATGGCGACTGAAGATGTGC
		APT1_R	TCAGTGTCGAGAAGAAGCGT
ATE1	*AT5G05700*	ATE1_FW	TCCTCTCCGTTTCCAGTGGG
		ATE1_R	CCACGAGAGTTTCAGAAGCACCAG
ATE2	*AT3G11240*	ATE2_FW	AGCAGTAGCAGAAACCGGAGTG
		ATE2_R	TTCTTGAACCGCGGTATATCCTTG
ETR2	*AT3G23150*	ETR2_FW	TGTTAGATTCTCCGGCGGCTATG
		ETR2_R	TTCCCATGAATCAACTGCACCAC
HRA	*AT3G10040*	HRA_FW	CATGACCAACAACCACCGCAAC
		HRA_R	TTCTGCTGCTGACTCGGAATCG
HRE1	*AT1G72360*	HRE1_FW	TCCGATGAGCCATTTGTCTTCTCC
		HRE1_R	CCATCTTCCCCAAGGCCTTC
HRE2	*AT2G47520*	HRE2_FW	TTGCTGCCATCAAAATCCGT
		HRE2_R	CCCCTGGTTTAGTATCGGCT
NR1	*AT1G77760*	NIA1_FW	CTGAGCTGGCAAATTCCGAAGC
		NIA1_R	TGCGTGACCAGGTGTTGTAATC
NR2	*AT1G37130*	NIA2_FW	AACTCGCCGACGAAGAAGGTTG
		NIA2_R	GGGTTGTGAAAGCGTTGATGGG
PCO1	*AT5G1512*	PCO1_FW	ATTGGGTGGTTGATGCTCCAATG
		PCO1_R	ATGCATGTTCCCGCCATCTTCC
PCO2	*AT5G39890*	PCO2_FW	TCCCCAGCCGAGTTCAGATA
		PCO2_R	TCCATCAGCCGGGTACAGTA
PDC1	*AT4G33070*	PDC1_FW	TCGATTGGGTGGTCTGTTGG
		PDC1_R	TGTCCTGAACCGTGACTTGG
PDC2	*AT5G54960*	PDC2_FW	TGAAAGCAATCAACACGGCA
		PDC2_R	CAGCAGAGACTCTAGAGCCC
PRT6	*AT5G02310*	PRT6_FW	CATATGGAGCCCTTGTTGCAGAG
		PRT6_R	TACACCAGTACCAGCACCACAG
RAP2.2	*AT3G14230*	RAP2.2_FW	CCTAGCGTCGTATCCCAGAA
		RAP2.2_R	CTCAGATGTGTTGGCTGCTG
RAP2.3	*AT3G16770*	RAP2.3_FW	AACTCACGGCTGAGGAACTCTG
		RAP2.3_R	ACGTTAACTTGGTTGGTGGGATGG
RAP2.12	*AT1G53910*	RAP2.12_FW	ACTGAATGGGACGCTTCACTGG
		RAP2.12_R	AGGGTTTGCACCATTGTCCTGAG
SRO5	*AT5G62520*	SRO5_FW	AAGAGGCGGTGCAGATGAAACAC
		SRO5_R	TTTCGAAACAGAGCACCAACCG
ALAAT1	*AT1G17290*	ALAAT1_FW	ATTCATGACAGATGGTGCAA
		ALAAT1_R	TATTTCAAGACCCCATCCTG
SUS4	*AT3G43190*	SUS4_FW	TTCACCATGGCTAGGCTTGA
		SUS4_R	CCACCAAGTTCACCAGTTCG
PGB1	*AT2G16060*	HB1_FW	GGCTCTTGTAGTGAAGTCTTGGA
		HB1_R	CTTCGTTGTTGGTGCAATCTCA
PGB2	*AT3G10520*	HB2_FW	TGAAGTCCCTCACAACAATCCTA
		HB2_R	AACGCCGCTTTTGAGATGAA
PGB3	*AT4G32690*	HB3_FW	TGGACGATTCGGTTGACATT
		HB3_R	TGGTTTATTGGCTGCGTGTT
HUP40	*AT4G24110*	HUP40 _FW	GAAACTTGAGTGCGAGTGTG
		HUP40 _R	CTCAAACCCAATCTTTTGCT
RBOHD	*AT5G47910*	RBOHD _FW	CTTCTGCAAACAAGCTCTCA
		RBOHD _R	GTATCCTGCTGTCTCCCATC
