# Supplementary material for: Ethylene-mediated nitric oxide depletion pre-adapts plants to hypoxia stress
Source: Nat Commun. 2019 Sep 5;10:4020. doi: 10.1038/s41467-019-12045-4 (PMC6728379; doi:10.1038/s41467-019-12045-4)
Supplement: Supplementary file 3 — Reporting Summary [file 41467_2019_12045_MOESM3_ESM.pdf]

nature research

Corresponding author(s): Rens Voesenek, Rashmi Sasidharan & Michael Holdsworth

Last updated by author(s): Aug 2, 2019

Reporting Summary

Nature Research wishes to improve the reproducibility of the work that we publish. This form provides structure for consistency and transparency in reporting. For further information on Nature Research policies, seeAuthors & Referees and theEditorial Policy Checklist .

Statistics

For all statistical analyses, confirm that the following items are present in the figure legend, table legend, main text, or Methods section.

n/a	Confirmed	
x	☐	The exact sample size (*n*) for each experimental group/condition, given as a discrete number and unit of measurement
☐	x	A statement on whether measurements were taken from distinct samples or whether the same sample was measured repeatedly
☐	x	The statistical test(s) used AND whether they are one- or two-sided<br>*Only common tests should be described solely by name; describe more complex techniques in the Methods section.*
☐	x	A description of all covariates tested
☐	x	A description of any assumptions or corrections, such as tests of normality and adjustment for multiple comparisons
☐	x	A full description of the statistical parameters including central tendency (e.g. means) or other basic estimates (e.g. regression coefficient) AND variation (e.g. standard deviation) or associated estimates of uncertainty (e.g. confidence intervals)
x	☐	For null hypothesis testing, the test statistic (e.g. *F*, *t*, *r*) with confidence intervals, effect sizes, degrees of freedom and *P* value noted<br>*Give P values as exact values whenever suitable.*
x	☐	For Bayesian analysis, information on the choice of priors and Markov chain Monte Carlo settings
x	☐	For hierarchical and complex designs, identification of the appropriate level for tests and full reporting of outcomes
☐	x	Estimates of effect sizes (e.g. Cohen's *d*, Pearson's *r*), indicating how they were calculated

Our web collection on statistics for biologists contains articles on many of the points above.

Software and code

Policy information about availability of computer code

Data collection	Image Lab 5.2<br>Neofox Viewer (Neofox Oxygen sensor)<br>Applied Biosystems ViiA 7 Real-Time PCR System (Thermo Fisher Scientific)
Data analysis	Applied Biosystems ViiA 7 Real-Time PCR System (Thermo Fisher Scientific)<br>Microsoft Excel 2010<br>Graphpad Prism 6<br>Icy imaging software v1.9.3.0 with ImageJ plugin v1.47<br>R version 3.3.4<br>RStudio 1.1.442<br>Image Lab 5.2<br>Neofox Viewer<br>Biorender

For manuscripts utilizing custom algorithms or software that are central to the research but not yet described in published literature, software must be made available to editors/reviewers. We strongly encourage code deposition in a community repository (e.g. GitHub). See the Nature Research guidelines for submitting code & software for further information.

Data

Policy information about availability of data

All manuscripts must include a data availability statement. This statement should provide the following information, where applicable:

- Accession codes, unique identifiers, or web links for publicly available datasets
- A list of figures that have associated raw data
- A description of any restrictions on data availability

Gene accession numbers of all the Arabidopsis genes/mutants/transgenics used in this study are listed in the Method section and Supplementary Table 1 and 2. Source Data related to Fig 1a, b; Fig 2a; Fig 3b, d, e, f; Fig 4a, b, d, e, f; Fig S1b, c, d, e; Fig S2a, b; Fig S3b, c; Fig S4a, b, c; Fig S5; Fig S6a, b, c, d, f; Fig S7a, b, c, d; Fig S8a, b, c, d, e; and Fig S10 are provided with the paper. No restrictions are placed on data availability.

Field-specific reporting

Please select the one below that is the best fit for your research. If you are not sure, read the appropriate sections before making your selection.

☒ Life sciences ☐ Behavioural & social sciences ☐ Ecological, evolutionary & environmental sciences

For a reference copy of the document with all sections, see nature.com/documents/nr-reporting-summary-flat.pdf

Life sciences study design

All studies must disclose on these points even when the disclosure is negative.

Sample size	No statistical methods were used to predetermine sample size. In general, the sample size of experiments was maximized and dependent on technical, space and/or time limitations. For survival assays, total sample size varied between between mutant lines/plant species and depended on germination rate where the mean of a row of seeds was used to determine survived/total plants. The maximum amount of rows and seedlings/root tips used per sample (biological replicate), generally 23 seedlings for Arabidopsis, is mentioned in the appropriate figure legends.
Data exclusions	No data was excluded after results were obtained. However, prior to experiments any in vitro grown plates containing seedlings were discarded if clear visual signs of bacterial of fungal infections were present. In addition, we excluded samples/mutant lines that germinated poorly. These experiments were redone using newly obtained seed batches.
Replication	Experimental repeats are mentioned in the figure legends. The majority of the experiments were replicated at least 3 times and showed similar results, independently of the investigator who performed the experiment. Additionally, some of the root tip hypoxia survival, RAP2.12 imaging and RAP2.3 western experiments were performed in more than one laboratory and yielded similar results.
Randomization	Plants or in vitro grown seedlings on agar plates were randomly assigned to treatment groups.
Blinding	Investigators were not blinded during experiments and outcome assessment.

Reporting for specific materials, systems and methods

We require information from authors about some types of materials, experimental systems and methods used in many studies. Here, indicate whether each material, system or method listed is relevant to your study. If you are not sure if a list item applies to your research, read the appropriate section before selecting a response.

Materials & experimental systems

n/a	Involved in the study	
☐	☒	Antibodies
☒	☐	Eukaryotic cell lines
☒	☐	Palaeontology
☒	☐	Animals and other organisms
☒	☐	Human research participants
☒	☐	Clinical data

Methods

n/a	Involved in the study	
☒	☐	ChIP-seq
☒	☐	Flow cytometry
☒	☐	MRI-based neuroimaging

Antibodies

Antibodies used	α-HA-HRP, High Affinitiy (3F10), Rat, Roche, Cat. No. 12 013 819 001<br><br>α-Actin (mAbGEa), Mouse, Invitrogen - Thermo Fisher Scientific, Cat. No. MA1-744

α-PGB1, Rabbit, Produced for this study using full length protein as antigen by GenScript

α-rabbit IgG-HRP, secondary ab for PGB1, Goat, Cell Signaling Technology, Cat. No. 7074

α-mouse IgG-HRP, secondary ab for actin, Horse, Cell Signaling Technology, Cat. No. 7076

Validation

The commercial primary antibodies were validated by the manufacturers (Roche, and Thermo Fisher Scientific).
The α-PGB1 was validated by co-author Hongtao Zhang, by Western Blot analysis on lysates of transgenic Arabidopsis line over-expressing PGB1 under a 35S promoter.
